# The role of isoprenoids in the chemical interaction between plants and other organisms in their rhizosphere

**DOI:** 10.1007/s42994-025-00225-4

**Published:** 2025-06-25

**Authors:** Harro Bouwmeester, Philipp Zerbe, Reuben J. Peters, Kangning Wang, Lemeng Dong

**Affiliations:** 1 Plant Hormone Biology Group, Green Life Sciences Cluster, Swammerdam Institute for Life Sciences, University of Amsterdam, 1098 XH Amsterdam, Netherlands; 2 Department of Plant Biology, University of California-Davis, Davis CA 95616, USA; 3 Department of Biochemistry, Biophysics, and Molecular Biology, Iowa State University, Ames IA 50011, USA

**Keywords:** Isoprenoids, Rhizosphere chemical interaction, Terpenoids, Strigolactones

## Abstract

Agriculture has become one of the largest users of non-renewable resources in the world and contributes heavily to resource depletion, environmental pollution, and climate change. Solutions to these problems are in dire need and these can partially be found in the inter-organismal interactions surrounding the rhizosphere of our crops. The rhizosphere is a highly complex ecosystem, serving as a habitat for a diverse array of beneficial and pathogenic organisms. Here, we review how plants are performing a balancing act, in which they employ chemical communication—through the exudation of chemicals from their roots—to recruit beneficial organisms, while keeping at the same time, pathogenic ones at bay. These metabolites released by roots are incredibly chemically diverse. Among them, isoprenoids, one of the most diverse metabolite classes, containing many, highly bioactive, molecules, are the focus of this review. A better insight into the chemical communication occurring between the root, the soil, and micro-organisms, will allow harnessing of the beneficial relationships and suppression of the harmful ones. Further, this will enable us to establish knowledge-based changes in how we perform agriculture, how we use chemical inputs, how we should breed more resilient crops and can bring back resilience to our agricultural soils.

## Introduction

Driven by the need to feed a burgeoning global population, agriculture has become one of the largest users of non-renewable resources in the world and contributes heavily to environmental pollution and climate change (Filonchyk et al. 2024). Agriculture causes environmental pollution through the use of chemical pesticides that accumulate in agricultural soils and surface waters, while the use of fertilizer is the primary source of the eutrophication of water bodies, primarily through the run-off of mainly nitrogen (N) and phosphorus (P) (Akinnawo 2023). Agriculture also contributes to climate change through CO_2_ and N_2_O emission, a substantial part of which is due to the use of chemical fertilizer. The production of N fertilizer, through the Haber–Bosch process, is one of the world’s largest energy consuming processes, and contributes substantially to global CO_2_ emission, while at the same time consuming large amounts of non-renewable fuel resources (Jacoby et al. 2017). Both the production and agricultural use of N fertilizer result in the emission of large amounts of NH_3_ and N_2_O, respectively (Charles Munch and Velthof 2007; Xu et al. 2024). The latter through nitrification of NH_4_^+^ to NO_3_^−^ (which can also yield N_2_O if incomplete) and denitrification of NO_3_^−^ to N_2_O (Charles Munch and Velthof 2007). The production of P fertilizer also relies largely on non-renewable resources, as it is mined as rock phosphate, a resource predicted to be exhausted within the next 100 years (Fayiga and Nwoke 2016).

As much as current agricultural practices exacerbate climate change, in turn, shifting environmental conditions have a severe impact on crop production as well as on pest and microbial diseases, which are tightly integrated with our agroecosystems (Savary et al. 2019). More frequent weather extremes and prolonged drought or flooding events reduce harvest yields, while pest and pathogen populations survive and thrive better under warmer conditions and can cause increased crop loss and mycotoxin contamination (Cheng et al. 2019; Yactayo-Chang and Block 2023). For instance, simultaneous stress caused by abiotic and biotic perturbations is predicted to result in up to 40% yield losses in major crops such as maize (*Zea mays*), rice (*Oryza sativa*), and wheat (*Triticum aestivum*) (Bailey-Serres et al. 2019; Savary et al. 2019). Faced with such yield impacts, use of pesticides and fertilizers increases, in turn, worsening the environmental footprint. As shifting environmental conditions can alter both cooperative and pathogenic plant–microbe interactions, it is critical to understand the molecular processes driving the above- and below-ground plant-environment communication.

Solutions for these problems are direly needed and evidence is accumulating in the literature that these can partially be found in the rhizosphere of our crops. The rhizosphere represents a highly complex ecosystem, serving as a habitat for a diverse array of beneficial and pathogenic organisms. Here, we review how plants are performing a balancing act, in which they employ chemical communication to recruit beneficial organisms, while keeping, at the same time, pathogenic ones at bay. Intriguingly, the organisms in the rhizosphere also communicate back to the plant, using chemistry. A better insight into the chemical communication, taking place belowground, will allow us to harness the beneficial relationships and suppress the harmful ones. And it will enable us to achieve knowledge-based changes in how we perform agriculture, how we use chemical inputs, how we should breed more resilient crops to restore resilience to our agricultural soils.

Plants invest significant amounts of carbon to recruit microbial partners, an indication of their reliance on such root microbiota. Many, often species-specific, secondary metabolites exuded by plant roots have been demonstrated to drive the assembly of the root microbiota (Pétriacq et al. 2017; Stassen et al. 2021; Tiziani et al. 2022; van Dam and Bouwmeester 2016). Indeed, it has been estimated that plants secrete from 5 to 30% of the carbon they fix in photosynthesis, into the soil (Sasse et al. 2018). The composition of the root exudate and its plasticity likely evolved to shape the root microbiota community composition to optimize plant growth, development and reproduction, under a range of different conditions (Lamichhane et al. 2024). Intriguingly, there is ample evidence that pathogenic organisms have also evolved the capacity to exploit root exudates to locate host plants (Kasteel et al. 2023). On the other hand, since modern, high-input, agriculture relies far less on the microbiome for nutrient acquisition and abiotic and biotic stress resilience, agriculture, and breeding may have resulted in genotypes that invest less carbon in the rhizosphere (Preece and Peñuelas 2020).

The root exudate is composed of a highly complex mixture of molecules from many different chemical classes that changes upon exposure of the plant to various stresses (Brisson et al. 2022; Tiziani et al. 2022). Many of the reported metabolites, obviously, represent relatively abundant chemicals and are comparatively simple, primary metabolites, that plants likely exude as carbon source for their root microbiota (Zhalnina et al. 2018). Others, usually specialized metabolites, exhibit anti-microbial activity and affect root microbiota composition through (selective) repression of some and (therefore) stimulation of others (Cotton et al. 2019; Stassen et al. 2021) or are considered signaling molecules providing information to and affecting the behavior of other (beneficial) organisms. These root exudate metabolites are incredibly chemically diverse, including phenylpropanoids, benzoxazinoids, oxylipins, alkaloids, and isoprenoids (Massalha et al. 2017). As one of the most diverse metabolite classes, containing many, highly bioactive, molecules, the isoprenoids are the focus of this review.

## Volatile terpenoids play essential defensive roles in the soil

Volatile isoprenoids are well known for their aboveground role in the chemical communication of plants with other organisms, such as pollinators and herbivores. However, these isoprenoids can also diffuse very well belowground, through the airspaces (Chiriboga et al. 2017) and can also play a role in chemical communication in the rhizosphere. The highly volatile monoterpenoids, for example, have been shown to also contribute to belowground plant defense mechanisms. For example, 1,8-cineole is produced in response to infection by *Pseudomonas syringae* in *Arabidopsis thaliana* (Arabidopsis) roots, as a defensive compound (Steeghs et al. 2004). Of the 32 putative terpene synthases (TPSs) in the Arabidopsis genome, 15 are primarily or exclusively expressed in roots (Vaughan et al. 2013). Moreover, the 1,8-cineole production from geranyl diphosphate (GDP) is catalyzed by two TPSs, both exclusively expressed in the roots, and the compound is only detectable in the rhizosphere, not in the roots (Chen et al. 2004).

**Fig. 1:**
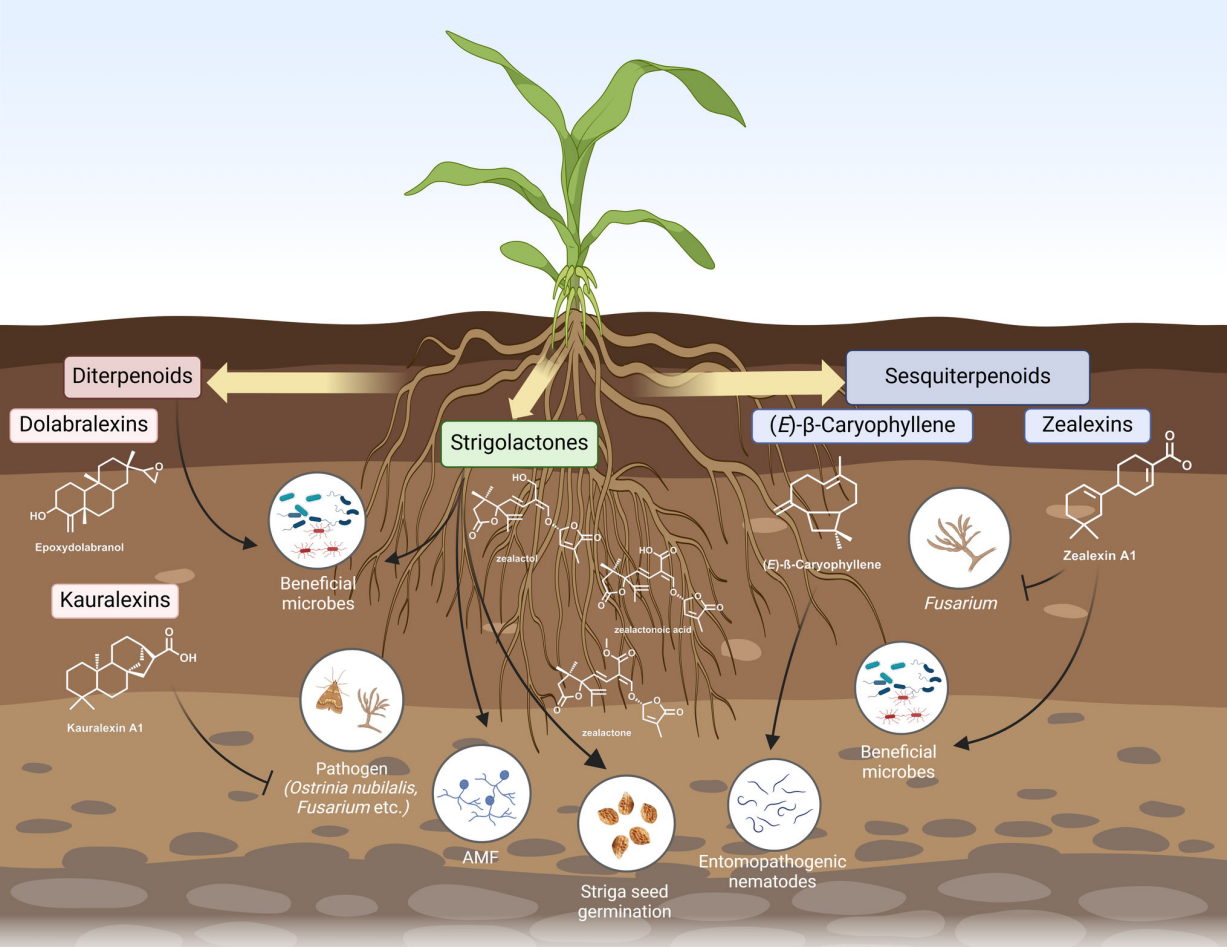
Chemical interaction between maize and other organisms within the soil. Through the elucidation of their biosynthesis and the generation of mutants, maize diterpenoids, such as the dolabralexins and kauralexins, have been shown to affect the microbiome and deter both pathogenic insects and fungi. The biosynthesis of maize strigolactones has also been elucidated and mutants showed that changes in strigolactone composition affect resistance against *Striga*, a parasitic plant. The effect of such changes on beneficial microbes and arbuscular mycorrhizal fungi (AMF) has yet to be studied. The volatile sesquiterpene, (*E*)-ß-caryophyllene, is an attractant of entomopathogenic nematodes that control corn root worm. The non-volatile sesquiterpenoids, zealexins, are inhibitory to *Fusarium*, whereas they stimulate beneficial microbes

In contrast to the induced production of 1,8-cineole, Arabidopsis constitutively emits rhizathalene A, a semi-volatile diterpenoid that is formed from the general 20-carbon precursor, geranylgeranyl diphosphate (GGDP), by the species-specific class I diterpene synthase (diTPS), TPS08, and contributes to belowground resistance against root-feeding insects (Vaughan et al. 2013). An example of indirect defense involves the sesquiterpene (*E*)-β-caryophyllene, which is released from maize (*Zea mays*) roots when fed upon by corn rootworm larvae (*Diabrotica virgifera virgifera*), attracting entomopathogenic nematodes, natural predators of the larvae (Köllner et al. 2008) (Fig. 1). Interestingly, the inoculation of maize roots with beneficial rhizobacterium, *Azospirillum brasilense*, increased the emission of (*E*)-β-caryophyllene, making it less attractive to the corn rootworm (Santos et al. 2014). Inoculation of maize roots with *Pseudomonas* had a priming effect, resulting in enhanced (*E*)-β-caryophyllene emission upon subsequent corn rootworm feeding (Chiriboga et al. 2018), possibly enhancing its attractiveness to entomopathogenic nematodes. Similarly, the *Citrus* rootstock can emit the homosesquiterpenoid, pregeijerene, upon infection with citrus root weevil larvae, *Diaprepes abbreviatus*, which was attractive to a number of different entomopathogenic nematode species (Ali et al. 2010).

Maize roots also exude the sesquiterpenoid, eudesmane-2,11-diol, in response to *Fusarium* infection (Liang et al. 2018), as a defense molecule, but possibly also as a signal to recruit beneficial, *Fusarium*-suppressing, microbes. In addition to the above volatile isoprenoids, the fungal pathogens also induce an array of other sesquiterpenoids in maize roots, such as the group of zealexin sesquiterpenoid acids (Huffaker et al. 2011) (Fig. 1). Zealexins are derived from the sesquiterpene (*S*)-ß-macrocarpene, formed by the macrocarpene synthases, ZmTPS6/Zx1 and ZmTPS11/Zx3, and downstream oxygenation by several members of the CYP71Z and CYP81A subfamilies of cytochrome P450 monooxygenases (P450s) (Ding et al. 2020). Quadruple insertion- and deletion-based mutants of the macrocarpene synthases did not display (inducible) zealexin biosynthesis and featured increased *Fusarium* susceptibility in stems and roots (Ding et al. 2020). The precise structure thereby impacts the antibiotic potency of different zealexins, as exemplified by zealexin A2, which represents a positional isomer in one hydroxyl group and lacks antimicrobial activity in contrast to zealexins A1, 3, and 4 (Ding et al. 2020; Huffaker et al. 2011) (Fig. 1). Moreover, zealexin biosynthesis is induced by drought and elevated CO_2_ concentrations, highlighting the role of maize terpenes in biotic and abiotic stress responses (Christensen et al. 2018; Vaughan et al. 2014, 2015). Costic acids form another group of acidic maize sesquiterpenoids, which are derived from α/β-selinene (formed by ZmTPS21), and confer pathogen and pest resistance in maize stems and roots (Ding et al. 2017).

The acyclic C11 homoterpene (*E*)-4,8-dimethyl-1,3,7-nonatriene (DMNT) has been frequently documented as a volatile compound induced by leaf herbivore attacks, attracting predators and parasites of these herbivores (Bouwmeester et al. 1999; Degenhardt and Gershenzon 2000). This DMNT is derived from the sesquiterpene (*E*)-nerolidol (Bouwmeester et al. 1999; Degenhardt and Gershenzon 2000). Interestingly, in *Arabidopsis*, DMNT is also emitted from roots in response to infection with *Pythium irregulare*, to which it provides protection. This DMNT, however, is synthesized through degradation of the non-volatile triterpenoid, arabidiol, rather than from (*E*)-nerolidol, an intriguing example of convergent evolution (Sohrabi et al. 2015).

The examples of belowground signals are quite scarce and this likely reflects their relatively challenging detection in soil, compared to aboveground. A combination of more targeted analysis of soil-borne volatiles, in combination with root gene expression analysis, will likely reveal that the volatiles reviewed here represent just the tip of the iceberg.

## Diterpenoids play multifaceted roles in plant chemical communication

The diverse group of diterpenoids has multiple physiological functions in plants, ranging from conserved gibberellin (GA) phytohormones contributing to plant growth and development to specialized, often species-specific, compounds that mediate allelopathy, various chemical defenses against herbivores, pathogenic microbes and nematodes, as well as abiotic stress responses and, possibly, beneficial root microbe interactions (Murphy and Zerbe 2020; Wang et al. 2023). GA pathway genes have served as a reservoir for repeated gene duplication and sub- or neo-functionalization events that gave rise to the vast expansion of specialized diterpenoids present in the plant kingdom (Zi et al. 2014). Functionally diverse families of diTPSs and P450s are the major drivers of diterpenoid chemical diversity, converting the central GGDP precursor into a vast array of different scaffolds and functional decorations (Banerjee and Hamberger 2018; Karunanithi and Zerbe 2019; Peters 2010). Beyond their catalytic diversity, functionally distinct diTPSs and P450s can interact, much alike LEGO building blocks, to form modular pathway branches that expand the product range (Jia et al. 2022; Karunanithi and Zerbe 2019; Peters 2010). As exemplified by the diterpenoid-metabolic network in maize, the presence of both catalytically redundant and promiscuous members, within these enzyme families, enables the biosynthesis of a large product repertoire while, at the same time, safeguarding against deleterious mutations in critical pathway nodes (Ding et al. 2019, 2020). Differential spatiotemporal and stress-elicited expression of core pathway genes can further mitigate dysregulation of primary metabolism and specialized defense pathways (Ding et al. 2019). For example, differential expression of the catalytically identical maize *ent*-copalyl diphosphate (CDP) synthases, ZmTPS37/CPS1/AN1 and the stress-inducible ZmTPS38/CPS2/AN2, ensures *ent*-CDP precursor supply to GA biosynthesis and the formation of stress-defensive diterpenoids, respectively (Ding et al. 2019).

**Fig. 2:**
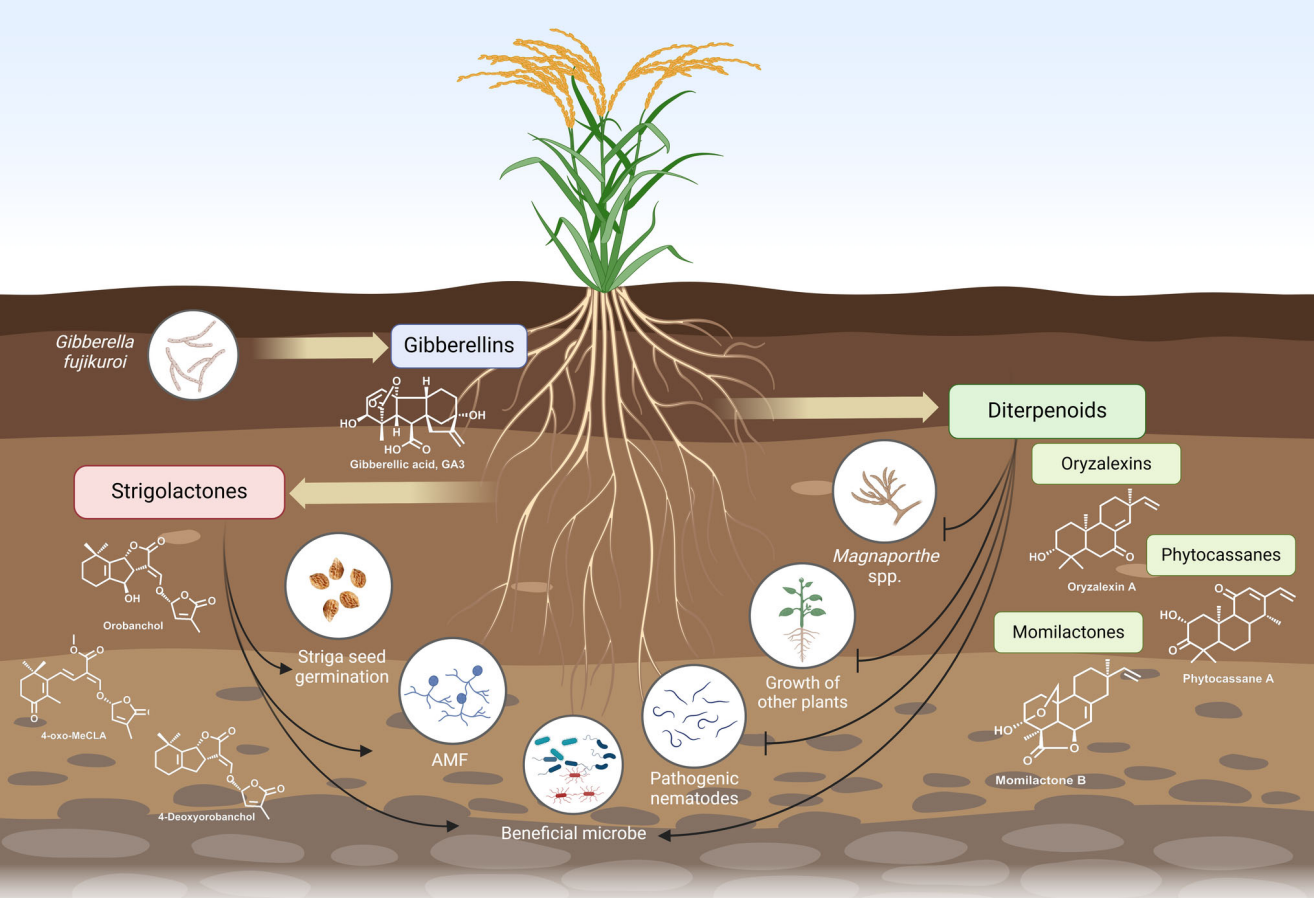
Chemical interaction between rice and other organisms within the soil. The fungus *Gibberella fujikuroi* produces gibberellins that affect rice growth. Rice strigolactone biosynthesis is largely resolved and studies with mutants demonstrate their role in the recruitment of beneficial microbes, including arbuscular mycorrhizal fungi (AMF), and are used as a host-presence cue by the parasitic plant *Striga*. Biosynthetic pathway elucidation and mutant generation demonstrated that certain diterpenoid oryzalexins, phytocassanes, and momilactones are inhibitors of pathogenic fungi and nematodes, as well as being allelopathic and promote recruitment of beneficial microbes

Extensive studies in rice have provided a detailed understanding of the dynamic network that controls the formation of diterpenoids, including oryzalides, oryzalexins, phytocassanes, and momilactones with functions in pathogen defenses and, for momilactones A and especially B, allelopathic interactions (Schmelz et al. 2014) (Fig. 2). Indeed, momilactones A and B were first discovered as plant growth inhibitors (Kato et al. 1973), and were later shown to be secreted from the roots (Kato-Noguchi et al. 2007). Their role in allelopathy was demonstrated by the loss of such activity from an insertional knock-out line for the relevant *syn*-CDP synthase *OsCPS4* (*cps4ko*), or subsequently acting *syn*-pimaradiene synthase *OsKSL4*, providing the first genetic evidence for this concept (Xu et al. 2012).

In addition to momilactones, rice also secrete phytocassanes from their roots (Toyomasu et al. 2008). Given their known importance in defense against both the bacterial pathogen *Xanthomonas oryzae* and fungal pathogen *Magnaporthe oryzae* (Li et al. 2022; Zhang et al. 2021), as well as the root-directed fungal pathogen *Magnaporthe rhizophila* (Lu et al. 2018), such secretion suggests that rice diterpenoids may mediate in more general interactions within the rhizosphere. Indeed, studies of CRISPR knock-out lines, generated in the model cultivar Kitaake, for not only *OsCPS4* (*cps4*) but also the inducible *ent*-CDP synthase *OsCPS2* (*cps2*), which is relevant to phytocassanes biosynthesis, as well as the *cps2* × *4* double mutant, demonstrate that rice diterpenoids also function in defense against the root-knot nematode, *Meloidogyne graminicola*, impacting not only resistance to this phytoparasite but also the composition of root and rhizosphere nematode communities more generally (Desmedt et al. 2022) (Fig. 2). Further analysis of these samples has demonstrated that these rice diterpenoids affect the microbiota of the rhizosphere and, especially, roots (Kudjordjie et al. 2024).

Like rice, maize produces several groups of species-specific diterpenoids, which serve as key components in warding off pathogens and pests at the site of attack (Schmelz et al. 2014; Yasmin et al. 2024). Alongside antimicrobial zealexin sesquiterpenoids discussed above, maize produces acidic diterpenoid phytoalexins, termed kauralexins, which serve as potent antibiotics against both pathogens and insect pests in above-ground tissues (Ding et al. 2019; Schmelz et al. 2011) (Fig. 1). Later work highlighted that kauralexins accumulate also in roots upon exposure to biotic and abiotic stressors, and a maize mutant deficient in the *ent*-CDP synthase, *Zmtps38/cps2/an2*, that controls the committed reaction en route to kauralexins, is more susceptible to drought along with featuring an altered root-to-shoot ratio (Vaughan et al. 2015).

Recently, dolabralexins were identified as an additional group of maize diterpenoids that are formed through the pairwise activity of ZmTPS38/CPS2/AN2 and the dolabradiene synthase, kaurene synthase-like 4 (ZmKSL4), and the P450s CY71Z16 and CYP71Z18 (Mafu et al. 2018) (Fig. 1). In several maize genotypes, dolabralexins represent the predominant metabolites in field-grown roots, and accumulate in and are exuded from roots following biotic and abiotic stress (Mafu et al. 2018; Murphy et al. 2021). Interestingly, the *Zmtps38/cps2/an2* mutant, lacking both dolabralexins and kauralexins, shows an altered rhizosphere microbiome in field studies (Murphy et al. 2021), suggesting a role for maize diterpenoids in root-microbiome interactions (Fig. 1). Indeed, a dolabralexin-deficient *Zmksl4* mutant displayed an altered root architecture (Murphy et al. 2023) and dolabralexins accumulate in plants grown in field soil but are largely absent in potting soil, suggesting that the presence of beneficial, or pathogenic, microbes affect below-ground diterpenoid accumulation (Murphy et al. 2023).

Studies in barley (*Hordeum vulgare*) identified hordedanes, as a group of antimicrobial diterpenoids that accumulate in barley roots in response to pathogen attack by *Bipolaris sorokiniana* and *Fusarium graminearum* (Liu et al. 2024). Strikingly, hordedane-deficient mutant lines—via loss of function in the involved diTPS genes, *Hvcps2* and *Hvksl4*—showed decreased colonization by *B. sorokiniana*, whereas *F. graminearum* was significantly increased. These findings demonstrated that hordedanes promote *B. sorokiniana* germination and growth, while suppressing that of other fungal pathogens, thus providing an intriguing example of microbial adaptation to plant chemical defense tactics (Liu et al. 2024).

Root diterpenoids and the underlying biosynthetic genes and pathways have also been identified in other crops, including wheat, millet (*Setaria* spp.) and switchgrass (*Panicum virgatum*). For instance, switchgrass produces panicoloids, a family of furanoditerpenoids that accumulate in root tissue in response to drought stress (Muchlinski et al. 2021; Pelot et al. 2018; Tiedge et al. 2022). Additionally, in vitro bioactivity assays, using metabolite extracts and fungal isolates (species of *Linnemannia*, *Trichoderma* and *Fusarium*) obtained from switchgrass roots, showed that metabolite blends of distinct ecotypes differ in their impact on microbial growth, with diterpenoids, including panicoloids, and triterpenoid saponins representing the major antibiotic compounds (Li et al. 2023b).

The above examples paint an emerging picture of multilayered functions of specialized diterpenoids far beyond well-established phytoalexin bioactivities that include roles in below-ground inter-organismal interactions with nematodes, fungal pathogens and chemical communication with the microbiome.

## Triterpenoids shape the root microbiome

Among the most abundant and structurally diverse families of specialized plant metabolites are the triterpenoids, which are broadly distributed across plant organs, with roots often producing species-specific types. Triterpenoids are synthesized via the mevalonate pathway, leading to the production of squalene, which is then cyclized and modified to form diverse triterpenoid structures.

The essential roles of triterpenes in shaping rhizosphere microbiota have garnered increasing attention (Zhalnina et al. 2018). Indeed, disruption of the biosynthetic pathway of three *Arabidopsis* root triterpenes, thalianin, thalianyl fatty acid esters, and arabidin, as well as treatment with these compounds, resulted in notable changes in root microbiota assembly (Huang et al. 2019). In cucumber and related crops the cucurbitacin triterpenes, which confer a bitter flavor, are involved in plant–microbe interactions (Shang et al. 2014). For instance, cucumber cucurbitacin B selectively enriches *Enterobacter* and *Bacillus* species, altering the rhizosphere microbiome, which then confers resistance to *Fusarium oxysporum* (Zhong et al. 2022). Similarly, glycyrrhizin, a triterpenoid saponin, modulates and enriches specific rhizospheric bacteria, particularly increasing the prevalence of taxa within the *Novosphingobium* genus or *Sphingomonadaceae* family (Liu et al. 2023). Ginsenosides, another special group of triterpenoid saponins, have also been shown to stimulate potential soil-borne pathogens and alter soil fungal community composition (Li et al. 2020).

In addition to shaping the population distribution of soil microorganisms, triterpenes from several plant species have been identified as direct stimulants for certain pathogenic organisms. For instance, ginsenosides have been shown to act as allelopathic stimulators, increasing the abundance of root rot pathogens, such as *Fusarium oxysporum*, as well as pythiaceous fungi like *Phytophthora cactorum* and *Pythium irregulare* (Li et al. 2020; Nicol et al. 2003). Similarly, Escaray et al. (2024) demonstrated that silencing lanosterol and butyrospermol synthase, in the triterpenoid pathway of *Euphorbia lathyris*, enhanced resistance to *Botrytis cinerea*, whereas the application of various plant saponins promoted the germination of *B. cinerea* spores.

**Fig. 3:**
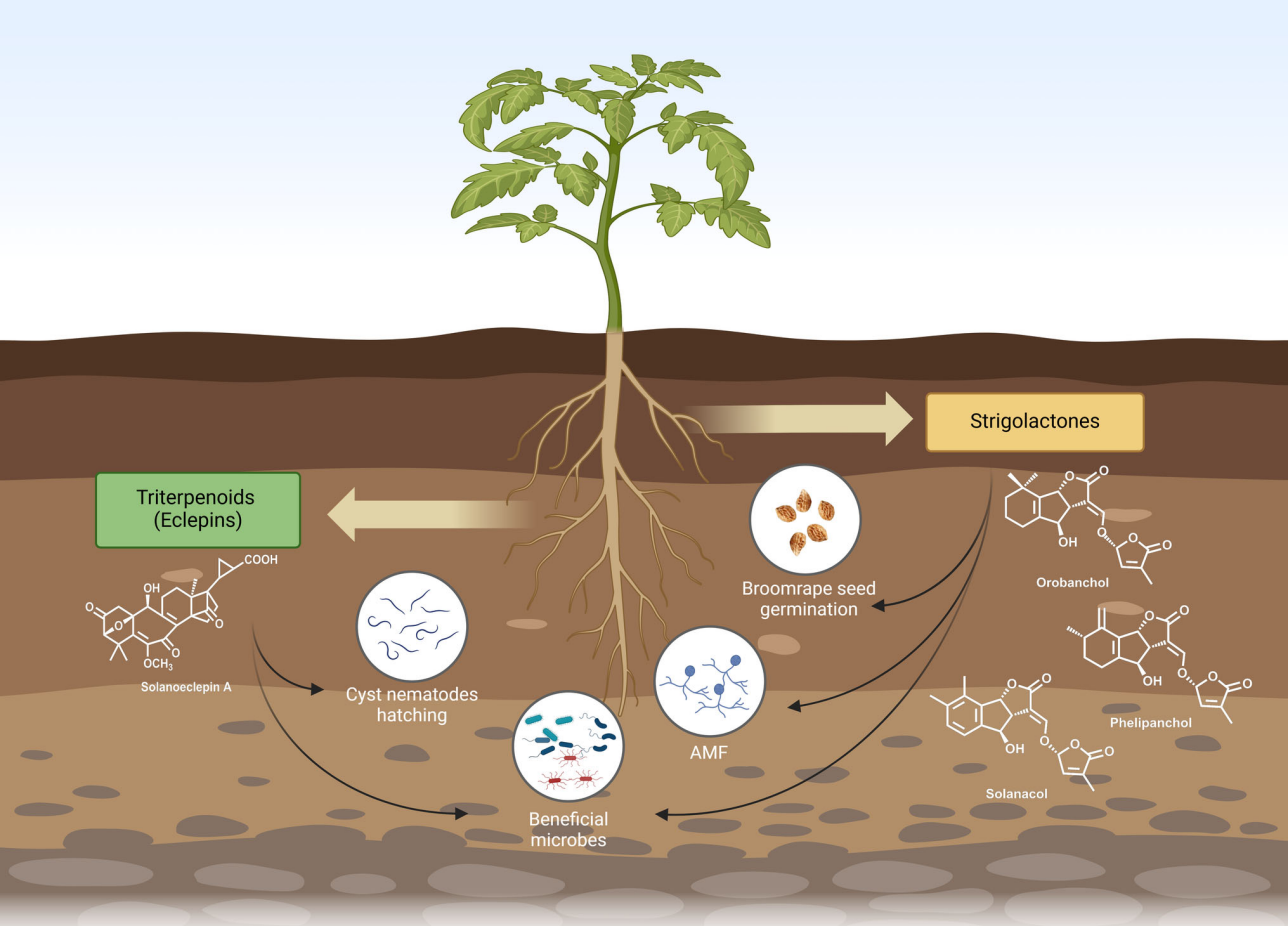
Chemical interaction between tomato and other organisms within the soil. Tomato exudes triterpenoid eclepins into the soil. These eclepins can induce hatching in cyst nematodes and appear to recruit beneficial microbes. The biosynthesis of strigolactones in tomato is largely resolved and several mutant studies have demonstrated their importance as cue for the parasitic broomrapes, whereas they also play a role in the recruitment of arbuscular mycorrhizal fungi (AMF) and beneficial microbes

Certain triterpeniods also act as hatching factors for cyst nematodes, mediating interactions between plants and these parasitic nematodes (Fig. 3). The first triterpenoid identified as a nematode hatching factor was glycinoeclepin A, a nortriterpene from the roots of kidney bean (*Phaseolus vulgaris*), which can stimulate the hatching of soybean cyst nematode, at concentration as low as 10^−11^ to 10^−12^ g·mL^−1^ (Masamune et al. 1982). Subsequently, glycinoeclepins B and C, with structures similar to glycinoeclepin A, were also isolated from kidney bean root extracts (Fukuzawa et al. 1985). Intriguingly, seventeen years after the identification of glycinoeclepin A, in tomato and potato root exudate, a triterpenoid with a structure quite similar to that of the glycinoeclepins was identified as the hatching stimulant for potato cyst nematode, and coined solanoeclepin A (SolA) (Schenk et al. 1999) (Fig. 3). Recently, SolA has gained significant attention due to its biological importance and complex, unique structure (Guerrieri et al. 2021; Shimizu et al. 2023; Sun et al. 2019; Vlaar et al. 2022). Intriguingly, recent work showed that SolA is likely the product of a microbial conversion of a plant produced precursor (Shimizu et al. 2023; Davar Abedini, personal communication). The latter is indirectly supported by earlier work demonstrating that incubation with rhizosphere microbes enhances the hatching-stimulant activity of the potato root exudate (Ryan and Jones 2003). Given the occurrence of eclepins in the root exudates of phylogenetically distinct species and considering that cyst nematodes exploit these compounds likely because they are reliable cues for host detection, it is plausible to hypothesize that eclepins, or their precursors, originally evolved for a positive and indispensable function, such as the interaction with beneficial microorganisms (Fig. 3). The fact that SolA biosynthesis is upregulated by N deficiency (Guerrieri et al. 2022) would provide additional support for this hypothesis.

An important biological role for the host of these eclepins would be an intriguing finding. The eclepins would then resonate the same principle as the strigolactones that are described below, of an essential host molecule that is exploited by plant enemies as cue for locating their plant host (Bouwmeester et al. 2021).

## Apocarotenoid strigolactones mediate the interaction with parasitic plants and microbiome

The first strigolactone, strigol, was discovered because it induced germination in the parasitic plant, *Striga lutea* (Cook et al. 1966). However, some 35 years later it was discovered that strigolactones are signaling molecules essential for root colonization by arbuscular mycorrhizal (AM) fungi (Akiyama et al. 2005), again showing that plant enemies, in this case the parasitic plants, use essential host signaling molecules as a cue (Bouwmeester et al. 2021). It was subsequently discovered that strigolactones are also a new plant hormone that controls branching in plants (Gomez-Roldan et al. 2008; Umehara et al. 2008). The strigolactones, as we know them, have all been identified in the root exudates of a variety of plant species and, so far, the structural resemblance between this branching inhibiting hormone to other strigolactones remains to be established.

Strigolactones are a signal for symbiotic AM fungi, which can solubilize complexed phosphate from the soil and exchange it with the plant in return for carbohydrates and lipids (Akiyama et al. 2005; Bouwmeester et al. 2021). The symbiosis of plants and AM fungi evolved about 450 million years ago and strigolactone production by plants seems just as old (Bouwmeester et al. 2021). Indeed, the extant primitive land plant, *Marchantia*, produces a strigolactone called bryosymbiol (Kodama et al. 2022). In higher plants, strigolactone biosynthesis evolved to a much higher complexity, with single species producing and exuding a blend of different strigolactones. The biosynthesis of a substantial number of these strigolactones has been elucidated (Bouwmeester et al. 2021; Huizinga and Bouwmeester 2023). Intriguingly, strigolactone biosynthesis is amazing complexity: ever more strigolactones are being discovered, with some 35 different structures known (Guercio et al. 2023). Furthermore, many different enzyme families have now been shown to be involved, with a carotenoid isomerase, two carotenoid cleavage dioxygenases, P450s from already four different subfamilies, one or two methyltransferases, an oxoglutarate-dependent dioxygenase, and a DIRIGENT protein (Homma et al. 2024; Huizinga and Bouwmeester 2023).

Studies with strigolactone mutants and genotypes exhibiting natural variation are slowly providing insights into the complex roles played these strigolactones in the rhizosphere. A mutation in the *Low Germination Stimulant 1* (*LGS1*) locus results in a change in the strigolactone composition, from mainly 5-deoxystrigol to orobanchol, in sorghum root exudate (Gobena et al. 2017), through as a yet mostly unknown mechanism (Shimels et al. 2024). These *lgs1* varieties induce much less germination in *Striga* and show significantly less parasitization by *Striga* in the field (Gobena et al. 2017; Mohemed et al. 2018). Also in maize and millet there is evidence that a change in the strigolactone profile—through a mutation, in both cases, in a methyltransferase—results in a certain degree of resistance to *Striga*, due to a much lower germination induction (Kuijer et al. 2024; Li et al. 2023a) (Fig. 1).

Surprisingly perhaps, the change in strigolactone profile in sorghum *lgs1* did not negatively affect the degree of symbiosis with AM fungi (Gobena et al. 2017). However, there were differences in the bacterial community composition, with the abundance of *Acidobacteria*, *Burkholderia*, *Cupriavidus*, *Acidovorax*, and *Albidiferax* being higher in the orobanchol-producing SRN39 (Schlemper et al. 2017). So far it is unclear whether this change has any consequences for host fitness, or perhaps it also contributes to *Striga* resistance (Shimels et al. 2024). A study in *Arabidopsis* with the strigolactone biosynthetic mutant *max4* also showed a difference in the microbial community composition upon the loss of strigolactone biosynthesis, but only for fungi (Carvalhais et al. 2019). In another study in rice, data integration was used to link strigolactones and the fungal and bacterial community in a series of genotypes displaying natural variation in the production of the strigolactones, orobanchol, 4-deoxyorobanchol and methoxy-5-deoxystrigol, as well as strigolactone biosynthetic and perception mutants (Kim et al. 2022) (Fig. 2). This demonstrated a positive correlation between especially orobanchol and the recruitment of AM fungi, but also with bacteria, including *Burkholderia*, which was also reported to be enriched in the orobanchol-producing sorghum (Kim et al. 2022; Schlemper et al. 2017; Shimels et al. 2024).

In a study on the transcriptional, root exudate metabolome and root microbiome responses in tomato to N deficiency it was shown that strigolactone biosynthesis was also induced under these conditions, just as in maize and certain legumes. Data integration with the microbiome analysis suggested that this increased strigolactone formation correlated with the enrichment of certain bacteria, potentially involved in N metabolism (Davar Abedini, personal communication). A *ccd8* mutant of tomato displayed little difference in its microbial community composition with wildtype; however, when grown under N deficiency, a number of microbiota, possibly involved in N metabolism, became differential, in that they were enriched in the wildtype (Fig. 3).

A picture is thus emerging that strigolactones are signals for several different mutualistic relationships, not just for the first reported arbuscular mycorrhizal fungi (Akiyama et al. 2005), as they are essential signals for plants to produce, and they serve as a reliable cue for parasitic plants (Bouwmeester et al. 2021). Specificity in the recruitment of these different mutualists, and avoidance of parasitic plants, is possibly achieved through strigolactones structural diversity, which would explain the large structural diversity in this class of molecules (Bouwmeester et al. 2021).

## Bidirectional communication: microbial isoprenoids

Although our focus is largely on how isoprenoids, exuded by plants, shape the microbiome of their rhizosphere, the microbes themselves also use such natural products to manipulate their hosts. The classic example indeed led to discovery of the gibberellin (GA) plant hormones, which were first identified in the rice root fungal pathogen, *Gibberella fujikuroi* (anamorph *Fusarium fujikuroi*), leading to their name (Macmillan and Takahashi 1968) (Fig. 2). GA biosynthesis is complex, requiring a minimum of 13 transformations, albeit several steps are accomplished by multifunctional enzymes in both plants and microbes (Hedden 2020). Consistent with GA serving as a secondary metabolite in *G. fujikuroi*, a corresponding biosynthetic gene cluster was identified (Tudzynski and Hölter 1998), which typically contains the seven genes necessary to produce the persistent bioactive GA_3_ (Malonek et al. 2005). Discovery of a mutant that no longer makes GA (Fernández-Martn et al. 1995) and was later shown to have lost the cluster (Tudzynski et al. 2001), enabled the demonstration that GA_3_ assists cell penetration by this pathogen during its root infection (Wiemann et al. 2013).

GA is also produced by certain nodulating, nitrogen-fixing rhizobacteria (MacMillan 2001). Again, the biosynthetic pathway is encoded by an operon, which is scattered throughout the rhizobacteria (Hershey et al. 2014), that generally contains eight to eleven genes (Nett et al. 2020), but only leads to production of the penultimate intermediate GA_9_ (Nett et al. 2017b). A small subset of these rhizobacteria also separately contain *CYP115*, which catalyses the final step and generates bioactive GA_4_, matching activity shown in plants—i.e., it acts as a GA3-oxidase (Nett et al. 2017a). Notably, a complete (*CYP115* containing) operon is present in a few bacterial phytopathogens, which then produce the bioactive GA_4_ themselves (Nagel et al. 2017). The operon also exhibits an intriguing, scattered distribution in the phytopathogens, for example in the rice pathogen *Xanthomonas oryzae*, whereas it is not present in the leaf blight pathovar *oryzae*. However, it is widespread in the leaf streak pathovar *oryzicola* (Nagel and Peters 2017), where the resulting GA_4_ seems to act as a virulence factor, suppressing the usual jasmonic acid defense response (Lu et al. 2015). Such suppression may underlie the loss of *CYP115* in the rhizobacterial GA operon, as expression of a GA3-oxidase in the nodule would remove the selective pressure for its retention and enable the legume host plant to exert more control over GA production. The effect of the resulting GA_4_ is to increase nodule size, which provides a strong selective advantage for the rhizobacteria (i.e., greater number of progeny), as well as for the plant, given that larger nodules fix nitrogen more efficiently (Nett et al. 2022).

Beyond GA, certain bacteria produce the prenylated nucleotide cytokinins (Mok and Mok 2001), which have been shown to serve as a virulence factor at least for *Rhodococcus fascians* (Pertry et al. 2009). By contrast, rhizobacteria seem to require distinct triterpenoid hopanes for free-living versus symbiotic states (Kulkarni et al. 2015). Otherwise, relatively little is known about the role played by microbially produced isoprenoids, particularly in their interaction with plants (Rudolf et al. 2021). However, the antibiotic activity exerted by these microbial natural products presumably shape their communities, and it can be speculated that this may lead to tritrophic interactions, wherein plants attract certain microbes to help shape their rhizosphere and root microbiota (Avalos et al. 2022). For example, the microbial terpene biosynthetic genes are enriched in the microbiota of plant roots infected by *Rhizoctonia solani* (Carrión et al. 2019).

Regardless, the extended evolution necessary for development of the complex biosynthesis of the gibberellins, which occurred independently in fungi and bacteria, emphasizes the strong selective pressure driving such isoprenoid metabolism in these plant-associated microbes, both symbiotic and pathogenic.

## Prospects

In the past two decades, research has rapidly expanded into the chemical communication occurring in the soil. With detection techniques and molecular tools becoming increasingly versatile and sensitive, the compartment of plants that was long hidden, the root, and the soil under its influence, the rhizosphere, has become accessible to plant scientists. This has led to the discovery of a chemical diversity that often surpasses that of the shoot. Such diversity is undoubtedly due to the complex interactions of plants with both beneficial and pathogenic organisms within the soil, which drove an arms race whereby specificity towards beneficial partners needed to be safeguarded while at the same time avoiding pathogenic organisms. On the other hand, current high-input agriculture has likely eroded the importance of this natural chemical communication, as nutrients and protection against pests and diseases are provided through chemical inputs. Indeed, comparing modern crops with their wild ancestors, or even across a chronosequence of crop genotypes, suggests that ‘bits and pieces’ of the chemical interaction with beneficial microorganisms may have been lost just because it was no longer required. With the increasing understanding of the events happening in and around plant roots, intervention to exploit these mechanisms for making agriculture more sustainable, is now coming into reach.

These interventions could entail the down regulation of the biosynthesis of isoprenoids that have a negative fitness effect on plants, in agriculture, as well as the upregulation of compounds with beneficial effects. The intriguing examples of the former are the selection for, or gene editing of, crop genotypes aimed at lowering their strigolactone exudation. Indeed, in several studies, strigolactone biosynthesis in tomato was targeted through gene editing. Disruption of strigolactone biosynthetic genes, *CAROTENOID CLEAVAGE DIOXYGENASE 8* (*CCD8*) and *MORE AXILLARY GROWTH 1*, as well as the strigolactone transporter, SlABCG45, decreased strigolactone exudation, which then resulted in field resistance to broomrape (Ban et al. 2025; Bari et al. 2019, 2021; Kohlen et al. 2012) (Fig. 3). The possible negative consequences of this engineering, on the recruitment of beneficial micro-organisms (Fig. 3), are generally ignored, except in a study that demonstrated colonization by arbuscular mycorrhizal fungi was slightly delayed in their *CCD8* RNAi lines (Kohlen et al. 2012).

Examples of the upregulation of beneficial compounds are scarce, as this is often more difficult to achieve through gene editing approaches. However, overexpression in maize of an (*E*)-β-caryophyllene synthase gene from oregano resulted in constitutive emission of this sesquiterpenoid (Degenhardt et al. 2009) (Fig. 1). In field assays, in the presence of corn rootworm and entomopathogenic nematodes, these (*E*)-β-caryophyllene-emitting plants suffered significantly less root damage and had 60% fewer adult beetles emerge than untransformed, non-emitting lines. However, in a subsequent study, it was shown that, despite the positive effect on the attraction of entomopathogenic nematodes, there were also costs associated with constitutive volatile production that overshadowed its benefits (Robert et al. 2013).

Hence, it is clear more knowledge is needed, as well as engineering approaches that can really fine-tune what we are trying to achieve. These efforts are imperative considering agricultural production that is increasingly challenged by detrimental climate conditions, environmental impact and rising prices for agricultural inputs that are deemed to make current agricultural practices unsustainable.

## Data Availability

Data sharing is not applicable to this article as no datasets were generated or analyzed as part of this study.
